# The Study of Medicine

**Published:** 1861-10

**Authors:** 


### MEDICAL CRITIC

AND

### PSYCHOLOGICAL

### JOURNAL.

OCTOBER, 1861.

### Art. I.—THE STUDY OF MEDICINE.

"When," says Archbishop Whately, "complaints are made—often not altogether without reason—of the prevailing ignorance of facts on such and such subjects, it will often be found that the parties censured, though possessing less knowledge than is desirable, yet possess more than they know what to do with. Their deficiency in arranging and applying their knowledge, in combining facts, and correctly deducing, and rightly employing general principles, will be perhaps greater than their ignorance of facts. Now, to attempt remedying this defect by imparting to them additional knowledge,—to confer the advantage of wider experience on those who have not skill in profiting by experience,—is to attempt enlarging the prospect of a short-sighted man by bringing him to the top of a hill. Since he could not, on the plain, see distinctly the objects before him, the wider horizon from the hill-top is utterly lost on him."

It would seem, indeed, as if everything had changed since the days of Bacon. Up to that time the prevailing fault among philosophers was hasty, careless, and scanty observation, and the want of copious and patient experiment. On supposed facts, not carefully ascertained, and often on mere baseless conjecture, men proceeded to reason, often very closely and ingeniously, forgetting that no architectural skill in a superstructure could give it greater firmness than the foundation on which it rests. In other words, they reasoned upon fancies instead of facts. Since the days of Bacon, there has been an ever-increasing tendency—

never greater than at the present day—to disparage the reasoning process, and to substitute in the place of this process "the mere accumulated knowledge of a multitude of facts," and to trust to what is often called *experience*, meaning by that an extensive, but crude and undigested, observation.

Now, we do not wish to be misunderstood in applying these general remarks to the present state of the medical sciences, for no one can be prouder of the triumphs of medicine, or less inclined to speak disparagingly of a profession which is only second to one in honour; but we would ask whether the difficulties which now beset the student are not rather in arranging and applying his knowledge, in combining facts, and correctly deducing and rightly employing general principles, than in any ignorance of facts? We would ask whether the multitude of facts which are now crowded into the different subjects with which the student of medicine has specially to do—anatomy, human and comparative, physiology, zoology, botany, chemistry, materia-medica, surgery, medicine, &c.—is not more than he knows what to do with, and whether in mastering these facts (if any one may hope to master them), there is not much danger of being bewildered, or, at best, of leaving little or no time for the deduction and right employment of general principles? That it is so, we, for our part, have no manner of doubt; and we are equally confident that this conviction is in no sense peculiar to us.

Sir William Hamilton has very happily shown that knowledge and intellectual cultivation are not identical—understanding by knowledge the mere possession of truths; by intellectual cultivation or development, "the power, acquired through exercise by the higher faculties, of a more varied, vigorous, and protracted activity."

"Knowledge and intellectual cultivation," he writes, "are not only not the same, but stand in no necessary proportion to each other. This is manifest if we consider the very different conditions under which these two qualities are acquired. The one condition under which all powers, and consequently the intellectual faculties, are developed, is exercise. The more intense and continuous the exercise, the more vigorously developed will be the power.

"But a certain quantity of knowledge—in other words, a certain amount of possessed truths—does not suppose, as its condition, a corresponding sum of intellectual exercise. One truth requires much, another truth requires little, effort in acquisition; and while the original discovery of a truth evolves, perhaps, a maximum of the highest quality of energy, the subsequent learning of that truth elicits, probably, but a minimum of the very lowest."

The neglect of this important distinction underlies and vitiates to no small extent the system of tuition pursued in our schools of medicine, and is the chief source of those evils to which we have referred. What, then, is to be done? Can any course be suggested which, while tending immediately to lessen these evils, would at the same time conduce to the better and fuller exercise of the higher faculties of the mind? We propose to indicate briefly one method, at least, in which we conceive it possible that these desirable ends may be obtained.

Enough has been already done to enable us to see that there is some fundamental principle at work in nature, and that the different phenomena of nature—those that are evidently normal as well as those that are apparently abnormal—will require to be interpreted by this principle before they can be fully understood. We do not say that this fundamental principle has yet been realized. We only say that enough has been done, not only to reveal the existence of such a principle, but to tell us *where* we may expect to find it. Now we would point out in what manner the search after this principle might aid the student in acquiring, eventually, something like fixed scientific principles. We remember well the difficulties of our own student days;—we remember the painful sense of bewilderment arising on all hands when beginning to collect our thoughts amidst the distracting whirl of phenomena, presented to our notice by lecture after lecture and sight after sight; we remember, also, with much thankfulness the instructions by which, after great turmoil and doubt, our mind was enabled to settle down towards something like settled scientific principles; and we venture to think that we shall not altogether err in seeking to direct attention to this subject.

In endeavouring to carry out our object it will be convenient to begin by asking a few questions concerning certain physical agencies which are of continually growing importance in medical studies. And first, of electricity. What is electricity? It is no single agent: it is a name for many agents in one—heat, light, magnetism, and others. What is magnetism? Nothing apart from electricity. What is artificial light? Like natural light, it goes hand in hand with heat, and it has the same power of working chemical wonders upon the magic screen of the photographic camera. What is heat? It is one of the signs of luminous and electrical and chemical action. What is chemical power? A power which bursts into light and heat in flame, and which changes into electricity and magnetism in the galvanic trough. What are the attractive forces which are associated with electricity and magnetism, and which play so important a part in chemical changes? Nothing is known about them, and, after all, they may prove to be only varying aspects of that force of attraction which is supposed to be neither electrical, nor magnetical, nor chemical—even the force of gravity. Indeed, so intimate and inseparable is the connexion between these agents, that it is more easy to look upon them as *signs of action* than as *agents*. It is impossible, indeed, to thoroughly investigate any one of these subjects without investigating all; and now, thanks to the labours of such men as Davy and Faraday and Grove, it is not difficult to see that there is here some general principle or law of which light, heat, electricity, magnetism, chemical power, and some kinds of motion, are only so many effects. It is not difficult to see that there is here some common principle among a multitude of facts.

But this is not all. It is not easy to draw a distinct line of demarcation between artificial light and natural light; and it is equally difficult to separate the phenomena which are correlative of artificial light from the phenomena which are correlative of natural light. Light, heat, and chemical power attend upon the force of gravity in the solar ray, and render it difficult to regard this force of gravity as an isolated and independent agent; and it is not easy to suppose that magnetism and electricity do not form part of the principle by which the earth and heavenly bodies are ruled. In other words, the province of astronomy becomes trenched upon, and the question arises whether the light and heat and *motion* even of the heavenly bodies are not to be regarded as mere signs of the working of one principle, and that principle the same as that which stands revealed to the eye of the experimental philosopher.

Certainly, it is more easy to believe in this unity of principle than to disbelieve in it—for the harmonious workings of nature appear to demand some harmonizing common principle—but it is much more easy to conceive the idea than to realize the grounds of such belief. At the same time, the attempt was made, not long ago, by an anonymous author in a book called "*Nomos*, or an attempt to demonstrate a Central Physical Law in Nature,"* and this attempt is well worthy of serious consideration. It is worthy of serious consideration, not only as pointing out with great clearness the common law which binds together electricity and light, and heat and the rest, but as reducing to one kind of motion the several kinds of movement which are the subject of ordinary experimental inquiry. In this work the author affords a physical explanation of those difficult motions which belong to electricity and magnetism, and not only so, but he attempts to show, that upon the same principle, the heavenly bodies will begin as well as continue to move, not only in their appointed orbits, but also

* Post 8vo. Longman. 1857.

around their axes. In a word, he attempts to show that the inorganic world is ruled by one single principle, of whose operation the phenomena of electricity, magnetism, light, heat, chemical action, and physical motion in every form, are only so many signs of action, and that no secret in the world of inorganic nature can be fully understood except upon this assumption of unity of principle or agent. Now such an attempt as this may be premature, and many of the conclusions may be incorrect, as in all probability they are, but the attempt, however imperfectly carried out, is one which claims the sympathy of every philosophical inquirer as a step made in the right direction.

There is, moreover, a certain connexion between vital and physical phenomena which would seem to show that all these phenomena may spring from a common cause, as indeed Mr. Grove has hinted at in his *Correlation of the Physical Forces.* The exercise of every function is attended with the generation of *heat.* The formation of carbonic acid and water in the act of respiration gives out latent caloric. The bodily temperature rises during inflammatory action and sinks during a period of fasting, and this without any corresponding variation in activity of the respiratory function. The bodily temperature rises equally during mental and nervous excitement, and sinks as constantly during the opposite periods of exhaustion and depression. *Chemical affinities* are called into play in respiration, and they are continually being detected in processes which were once thought to be exclusively vital in their character. *Electricity* is developed during the germination of seeds, as was first shown by Pouillet, and in the Gymnotus this development is so considerable that the creature may be said to be armed with mimic thunderbolts. *Light* is emitted by many fungi in certain states of the atmosphere, and by the hosts of marine animals which are seen to blaze in the eddy of a vessel. Nor is it different with the *motion* which is manifested in plants and animals, for there is reason to believe,* not only that vital and physical motion are connected, but that they are obedient to the same law. But it is in the periodical changes in the vital manifestations of plants and animals, and in the obvious dependence of these changes upon certain physical causes, that the intimate connexion between vital and physical phenomena is most clearly revealed ; and it may be well, therefore, to dwell upon these facts for a moment.

The periodical changes in the life of the sensitive plant are both plain and simple. In spring the seedling emerges from the

---

* See, in reference to this question, Physiological Introduction to Dr. Radcliffe's work on *Epileptic and other Convulsive Affections of the Nervous System.* Post 8vo. Churchill. 3rd edition. 1861.

cradle in which it had slept during the winter; in summer it puts forth its foliage; in autumn it droops; in winter it dies. In spring it gives new signs of life; in summer it regains its verdure; in autumn it fades; and in winter it again becomes a bare and lifeless twig. Year by year these phenomena succeed each other with unfailing regularity, and the vitality ebbs and flows in direct relation to the ebbing and flowing intensity of the sunbeams. At daybreak, also, the leaves recover from the closed and pendant condition in which they had been all night, and if not disturbed in any way, they remain unfolded till evening, when they again close and droop; and these changes alternate with perfect regularity so long as the leaves retain their characteristic irritability. In a word, these vital movements of the plant correspond to certain changes in the relative position of the earth and sun, the one referring to the annual, the other to the diurnal revolution.

And so also with the periodical changes which are exhibited in the life of the common newt. Like the seed of the plant, the egg exhibits no sign of development unless it be quickened by the sunbeams; and like the plant, also, the liberated animal is throughout its life dependent upon the same fostering help. As spring advances it grows day by day into a more active and sentient being; as autumn wanes it droops by degrees until at last all its faculties are locked up in unbroken sleep. This winter-slumber passes off at the return of spring, and the creature lives again until the end of autumn; and these changes are repeated through succeeding springs and autumns with as much regularity as the corresponding changes in the life of the sensitive plant. In summer, also, the newt wakes in the daytime, and sleeps at night. In a word, the life of this creature appears to be as closely wedded to the sun as is the life of the sensitive plant; and yet this life embraces a sentient principle which is endowed with memory and other mysterious gifts.

These changes are also reflected in other plants and animals. The woods and fields of this country are bare and desolate in winter, and the few trees and plants which retain their verdure are half dead, because the sun has withdrawn his warmer rays. The shores of Lapland present only scanty patches of moss, and the banks of the Amazon are hidden under an impenetrable and unbroken tangle of forest trees, because the arctic regions do not share in the perennial summer of the tropics. A brief winter may even be said to reign during the night, for on passing within the polar regions the seasons of winter and night are found to become confounded and identical.

The animal world exhibits the same obedience to the seasons. The herring which had been born in the northern seas during the summer forsakes its birthplace before the winter, and follows its parent luminary towards the south. The frog sleeps soundly under the waters of the frozen pond. The swallow shuns the winter, or if she remain, she forgets her solitude and sleeps until the chirp of her old companions is borne upon the warm gales of returning spring. The bat has lost its birdlike energy and it sleeps while the swallow is away. The marmot is only nimble during the summer, and at other times it must sleep or follow the day in its southward course. The animals, also, which live in the winter are still the subjects of the same law, for though they are clothed in warmer vestments, they have lost that vital heat, the overflowing of which provides for the renewal of their own life in that of their offspring.

The diurnal changes in the life of the newt are also reflected by diurnal changes in the life of other animals, and, as a rule, sleep attends upon the night and wakefulness upon the day. And that sleep is caused by the night and wakefulness by the day, may be argued from the changes by which the periods of sleep and wakefulness are made to correspond at all seasons to the continually changing periods of day and night. The sheep, for example, sleeps from sunset to sunrise, and wakes from sunrise to sunset, and this it does throughout the year. It cannot sleep, therefore, because it is *exhausted* from having been awake, for the slumber is briefest and lightest after the accumulated fatigue of a midsummer day, and longest and heaviest when winter has most abridged the period of daily exertion. The times of renewal and waste are, indeed, inversely related to each other. But the slumbers may be expected to observe the law which governs them, and be brief and light in summer and long and profound in winter, if the energy of waking life be derived from the sun, for then the animal *must* wake in the daytime and sleep at night, and the periods of wakefulness and sleep *must* bear an exact correspondence to the changing periods of day and night. The fact, therefore, that there are these changes and correspondences, and that sleep cannot well be accounted for as a consequence of the exhaustion of wakefulness, or wakefulness as a mere consequence of the refreshment connected with sleep, is a powerful argument that the sheep, and with it the great multitude of living creatures, wake and sleep in implicit obedience to the rising and setting sun.

Arguing from a fact connected with the "noctural life of animals in primeval forests," which forms a brilliant passage in the "Aspects of Nature," it would even seem that the moon has some share in the sleep-dispelling power which belongs to the sun. Humboldt writes:—"Soon after eleven o'clock such a disturbance began to be heard in the adjoining forest, that for the remainder of the night all sleep was impossible. The wild cries of animals raged through the forest; and among the many voices which resounded together, the Indians could only recognise those which, after short pauses in the general uproar, were first heard singly. There was the monotonous howl of the howling monkeys, the plaintive, soft, and almost flute-like tones of the small sapajous, the snorting grumbles of the striped nocturnal monkeys, the interrupted cries of the great tiger, the cuguar or maneless American lion, the peccary, the sloth, and a host of parrots or parraguas, and other pheasant-like birds. When the tigers came near the edge of the forest, our dog, which had before barked incessantly, came howling to seek refuge under our hammocks. Sometimes the cry of a tiger was heard to proceed from amidst the high branches of a tree, and was in such case always accompanied by the plaintive piping of the monkeys, who were seeking to escape from the unwonted pursuit." This extraordinary turmoil, it must be observed, occurred when "the night was humid, mild, and *moonlit;*" and the Indians accompanying Humboldt accounted for it as a consequence of the moonlight, saying that "the animals were rejoicing in the bright moonlight, and keeping the feast of the full moon." Humboldt himself attempts to account for it by ascribing it to some accidental combat—"the jaguar pursues the peccaries and tapirs, and these, pressing against each other in their flight, break through the interwoven tree-like shrubs which impede their escape; the apes on the tops of the trees, being frightened by the crash, join their cries to those of the larger animals; this arouses the tribes of birds which build their nests in communities, and thus the whole animal world becomes in a state of commotion;" and this may be the explanation occasionally. But the frequent connexion of the turmoil with full-moon light is curious; and this is the question of interest here. Is it that the nocturnal riot on the banks of the Cassiquiare, so vividly described, when the wild animals are "keeping the feast of the full moon," and the baying of the mastiff in the English homestead on the same occasion are parallel phenomena? for if they are, then the two facts afford a double reason for supposing that the moon is endowed with some degree of the vivifying power which belongs to the sun.

There are, also, certain familiar facts which serve to show that artificial light and heat have a sun-like influence upon plant and animal. The convolvulus awakes throughout the night in a well-lit room; the sensitive plant lives during the winter under the fostering shelter of the hot-house; the imprisoned squirrel does not hybernate in the warm kitchen; and the snail sleeps soundly through the summer in an ice house. These facts are of great interest in themselves, but they are of greater interest as confirming the conclusions which have been arrived at respecting the vivifying power of the sun, and as rendering it more than probable that the moon has some share of a similar energy; for if this vivifying power belongs to artificial light and heat, it is impossible to conceive that it does not belong to natural light and heat.

It is clear, however, that the sun and moon are not to be regarded as the sole spring of life, for there are animals which sleep in the daytime and wake at night, and there is man himself, who sleeps and wakes with little apparent regularity. And so it should be.

In the case of man, the periods of sleep and wakefulness must occur at irregular intervals, because there is no regularity in the times of meals, or in the various moods of pain and pleasure. Food supplies fuel, by the burning of which the Laplander is able to keep himself warm throughout his sunless winter. It feeds a fire within which does the office of the sun, so far as he is concerned; and what it does for him it does for all. If, therefore, the food be taken irregularly, the warming and vivifying effects resulting from that food must occur irregularly. Again, pain depresses and pleasure excites; and as pain and pleasure are events which occur without any definite order, there can be no definite order in the states of depression or excitement resulting from them. And certainly the pain of hunger is for a while sufficient to drive away sleep. In man, therefore, the mere irregularity which there is in the times of meals, and in the moods of pain and pleasure, is sufficient to account for the want of absolutely fixed periods of sleep and wakefulness.

On the same principles, it is easy to understand how some animals should wake when others are asleep. The animals which thus wake are supplied with richer food, and gifted with acuter feelings, than diurnal animals; and these facts may have a direct bearing upon the difference in their habits. If food exerts so decided an influence upon man, it may be expected to affect them similarly. If hunger wakes him, it may be expected to wake them. If, then, first of all, it be supposed that these animals were set to discharge their appointed duty at night, and that they were provided with appetites to devour a quantity of food sufficient for the wants of twenty-four hours, their hunger would wake them regularly at night, just as it wakes the man who has gone dinnerless or supperless to bed; and as they are devoid of any spontaneous power by which they could alter their relations to that law of which they are the subjects, they would continue to eat and wake and sleep in the same order. And that this is at least a part of the true explanation may be argued from the fact that there is a complete revolution in the habits of lions, and other nocturnal animals, by which they are made to wake in the day and sleep at night, when they are confined in menageries and fed in the daytime.

In spite of every cause of perturbation, there is a strong bond of connexion between sleep and darkness, even in man. It is something more than accident which so often causes the shepherd boy to sleep and wake with his flock. It is something more than accident which causes man to be stunted, stolid, and passionless in countries where the sun never rises for months together.

It is no part of our intention to pursue any speculations of this kind into particulars; but if it were otherwise, we should wish to remove all objections, and show that it is not possible to explain the periodicity of vital phenomena unless upon the supposition that the law which regulates these phenomena is one and the same with that law which rules, not the earth only, but the universe. Nay, we would wish to show that the *bodies* even, in which vital and physical phenomena are manifested,are connected in a way which cannot be understood, except upon the theory of some common archetype—an archetype which embodies every diversity of form, and connects not only the different parts of plants and animals with each other, but which connects plants and animals, and links the lower forms of organic life, and, through them, the higher, with inorganic bodies—with bodies which, in one sense, may be said to hold the relation of skeleton to the skeleton of plants and animals. These topics are indeed tempting, and we would fain dilate upon them, but we must be content with asserting that there need be no doubt upon the mind of any one who will yield himself to the patient investigation of nature, with such light as is already at his disposal, as to the actual existence of some grand, some fundamental principle in all phenomena, vital and physical, terrestrial and cosmical.

Now if there be such a principle as this, it is evident that all natural phenomena must be viewed in relation to it, and that no proper interpretation of any phenomenon can be hoped for in any other way. As well might we attempt to understand the falling of a stone towards the earth, or of the earth towards the sun apart from the law of gravitation. If there be such a principle it is no question of theory: it is one of practice—of every-day practice, and this will become apparent just in proportion to the degree in which medicine shall become raised to the rank of an exact science.

For what is disease? Is it something *unnatural*, or is it a *natural* consequence of the violation of some law or principle—of some altered relations of things in themselves natural and fixed? Do not the history of medicine and the spirit of philosophy alike require us to believe that it is something *natural*

and not something *unnatural*, and that disease has to be cured by removing the patient out of that state or position in which the disease is *natural?* Are not ague and remittent fever and dysentery, are not fevers and plague the *natural* consequences of the localities in which they arise, and may not these diseases be expected to disappear when these localities are changed? Of this there can be no doubt. And is it not so with cholera? Look at India, and it will be seen that ague, and remittent fever, and dysentery and *cholera* all originate in very similar localities, and under very similar circumstances, appearing as the water dries up from the swampy grounds and disappearing as the rains return. There is evidently the same cause at work, for not only does cholera show its connexion with what is called *miasm*, by appearing and disappearing with diseases which are so certainly connected with miasm as ague, remittent fever, and dysentery, but it bears witness to the same fact by interblending in many different ways with these very affections. This is no new opinion. On the contrary, the intimate connexion in nature and cause between cholera and ague, and between cholera and tropical fevers and dysenteries, is now very generally recognised by the persons best acquainted with tropical diseases. And certainly there is some reason to believe that the history of cholera in India is the history of cholera elsewhere, where that history is fully understood. At any rate, it is only by this history in India, as it seems to us, that the history of cholera in the metropolis in the last epidemic is to be understood. Did not the unusual prevalence of ague (a well-observed fact) before the outbreak of cholera bear testimony to the presence of some *miasm*, and did not the unusual dryness of the weather before and during the epidemic predispose to the generation of this miasm by allowing the mud in the drains and on the surface to pass into a state of perilous dryness? Interpreted by the history of India it was no anomaly that a pestilence so dire should stalk abroad under skies of such unusual brightness and serenity. No doubt, much has to be learned as to the particular nature of cholera, and as to the grounds upon which this affection differs from and agrees with ague and remittent fever and dysentery, but in the meantime something is learned which is not learned on the table of the dead-house, or in the field of the microscope, or by random trials of various remedies. Thus, if unusual dryness of the season (*i.e.* a tendency to tropical drought) be a cause, it is obvious that we should endeavour to reproduce as far as possible the state of things which obtains in rainy weather; and in low districts like Westminster, for example, where the water-supply is scanty at best, and altogether wanting in some parts, it becomes a question whether the tide ought to be altogether excluded from the drains in seasons of drought, and whether the best means of preventing or suspending a visitation of cholera would not be to open, occasionally and partially, the *pen-stocks* and *tide-flaps* at the mouths of the great drains. A plan of this kind was adopted, if we remember rightly, some years ago, after cholera had broken out in a town near the Ouse, in the East Riding of Yorkshire, and with immediate and permanent benefit. Or the requisite supply of water might be obtained from other sources. In any case, the object must be to take care that as much water enters the drains, or lodges upon the surface, as enters or lodges in comparatively wet weather. Again, if cholera be akin to ague and remittent fever and dysentery in its causes, it is obvious that quinine will be an all-important remedy, and especially as a prophylactic—an inference which is now being rapidly confirmed by experience; or at any rate, that light as to the treatment of cholera will be reflected from what is known—and not a little is now known—of the treatment of the affections akin to cholera.

Again, is there not reason to believe that consumption is something *natural*, and not something *unnatural*, and that this disease has to be cured by removing the patient out of that state or position in which it is *natural*? Look at the history of consumption in Scotland and Holland, as related by Dr. Beddoes. While the Scotch dressed in homespun linsey-woolsey and were warmly clad, and while the Dutch wrapped themselves until they made themselves more like bobbins than anything else, coughs were rarely heard in the churches of Scotland and Holland, and consumption was a rare disease; but when the Scotch adopted the thin cold cottons of Glasgow, and the Dutch donned the light French style of dress which is now the *mode*, their coughs began to disturb divine worship as much as they do now, and consumption became a common disease. Look again at butchers, who, as their ruddy appearance testifies, eat large quantities of animal food, and are, as a body, free from consumption; and remember the other day that some curious evidence was adduced to show that consumptive persons, as a rule, are in the habit of abstaining from fat. Remember, too, that a fluid fat—cod liver oil—has been found to be the most effectual remedy in the treatment of consumption. In other words, there is some reason to believe, that consumption may be the natural consequence of the surface being imperfectly protected in cold weather, so that, in common language, the blood has been driven to the lungs, and of certain errors of diet, by which too little animal food and too little fatty matters have been supplied habitually. These reasons, of course, among others, and many others. And certainly there is nothing in the diseased product called tubercle which need be any objection to this view, for this product is nothing more than certain natural products which have stopped short at a period of development, more or less rudimentary.

These are of course only hints, and very imperfect hints, of the way in which it may be expected that certain diseases will be shown to be something *in order*, and not something *out of order*. Demonstration is a different matter, and, perhaps, the time has not yet arrived in which demonstration may be hoped for; but we see enough already to be convinced that the time will come when every disease, acute and chronic, will be shown to be something as perfectly *in order*—something as perfectly *natural*—something as perfectly physiological—as health itself.

But once more to leave special speculations and return to the course which must be pursued in the search after principle or law, for we are yet far from the grandest generalization of all.

Now there is much that may be interpreted by an appeal to nature, and that will be interpreted just in proportion to the degree in which the person appealing is convinced of that unity of law to which we have been directing attention; but there is also much which cannot be interpreted by this means alone.

If we appeal to nature and ask what is life, and conscience, and will, and intelligence, with its wondrous power of memory and anticipation, what is the answer? The oracle is as dumb as that of ancient Delphi, for it boots nothing to be told to ransack the convolutions of the brain, and it avails as little to pore over the pages of the metaphysician. And yet the physician must set himself to learn all he can respecting these faculties, or he will not have much chance of relieving the most important maladies of which the human frame is subject.

But these faculties are not altogether unintelligible. They are unintelligible if information respecting them be sought solely in the oracle of nature; but they are not unintelligible if information be sought first at an oracle which is higher than that of nature. The former oracle is dumb: the latter answers simply and unequivocally that man was created in the image of Him who is revealed to us as eternal, infinitely holy, all-powerful, all-wise—even God himself. And if this, then, be the fact, is there any wonder that man, whose nature is thus Godlike, should be endowed with life and conscience, and will and intelligence? Assuredly not. The wonder is, not that man should *live*, but that he should be so shorn of the life of Him who is from everlasting to everlasting. The wonder is, not that man should be endowed with *conscience*, but that that beam which tells of the holiness of man's august original, should be so dimmed with evil. The wonder is, not that man should have a *will*, but that the image of the omnipotent God should be so uncertain and helpless. The wonder is, not that man should know a little, and remember a little, and anticipate a little, but that he should retain so little of the intelligence of Him to whom past and present and future are at once open. The wonder is, that man should be so "lapsed in time and passion"—but why talk of wonder, for if we listen to the same oracle which tells us that man was created in the image of God, we shall soon learn that man has forfeited his high birthright.

Now we believe, and we believe unhesitatingly, that it is only by accepting this key that we can hope to comprehend the higher mysteries of the life of man ; and we believe, moreover, that without this key it will be impossible to comprehend any vital phenomena whatever. Man is not altogether severed from the lower forms of life. On the contrary, there is a common type, a common law which binds him to them in an indissoluble alliance ; and hence it may be expected that the image of man will be reflected in them just as the Divine image is reflected by him. And if so, then it is no wonder that there should be manifold evidences of life and intelligence around and beneath man, and that these evidences should be *perfect* in their degree, because the lower forms of creation are not revealed to us as having partaken in the fall of man, or at any rate they are not revealed to us as having fallen in the same manner. And if the lower forms of creation have not fallen in the same sense that man has fallen, it is to be expected that the several vital phenomena which belong to these forms should be perfectly adapted to the conditions in which the forms are placed, and that any intellectual phenomena should partake of the character of *instinctive acts.* Indeed, all vital phenomena, high or low, must take place with perfect regularity under the same circumstances, because there is no perverted will to mask the manifestations of that perfect law of which the vital phenomena are the signs.

We believe, indeed, and we believe unhesitatingly, not only that there is no discrepancy between the teachings of Holy Writ and the teachings of nature, but we believe also that the mystery of nature will remain a mystery until philosophers will consent to listen to the teachings of Holy Writ. It is not enough to seek after a common law in the way in which it may be sought after in nature alone ; but it is necessary, even as a scientific question, to pursue the subject beyond—far beyond—and to do this with a view to know the Lawgiver as well as to comprehend the law. "A little philosophy," says Bacon, "inclineth man's mind to atheism; but depth in philosophy bringeth men's minds about to religion." It is not possible, indeed, to ignore religion in scientific inquiries, and to attempt to do so is to forego the possibility of attaining to the highest generalization—a generalization which gathers into one principle all phenomena, vital and physical, and which connects that principle with a guardian Deity in whom we live, and move, and have our being.

> " A pagan, kissing, for a step of Pan,
> The wild-goat's hoof-print on the loamy down,
> Exceeds our modern thinker who turns back
> The strata—granite, limestone, coal, and clay—
> Concluding coldly with—'Here's law! where's God?'"

So says the late accomplished author of *Aurora Leigh* of the modern thinker in geology; but if this be true of him—and who can doubt it?—how much more true must it be of the modern thinker in medicine.

We have chosen to dwell upon these topics—even at the risk of provoking a feeling of dissatisfaction in the minds of some of our readers—because we are firmly convinced that much of the present uncertainty in medicine must continue until medical men set themselves seriously to realize law, or principle, in nature. It is not, it cannot be enough to exercise the powers of observation, and to indulge in a casual reflection now and then; and it surely must be necessary to exercise the thoughts as much as the eyes and fingers. Far be it from us, however, to discourage observation, and to depreciate diagnosis and mechanical skill; on the contrary, we would excuse no shortcomings in these respects, but we would require that the powers of thinking should be cultivated in a systematic manner, eventually, perhaps, by a definite course of lectures, in which the object was to set forth the various arguments by which the unity of law in nature could be demonstrated.* We know it is no light thing to multiply the labours of the already overworked student, but we believe that a few lectures might be so arranged as to direct him to, if not to put him in possession of, a principle, of which even the knowledge of its existence would materially help him in mastering his profession. We believe, moreover, that a course like this would, if carried out honestly, be the means of counteracting, *on scientific grounds*, those mechanical and sceptical notions which have practically divorced religion from the thoughts of the student, and left him to grope his way, without any other guide than that of his own blinded reason, among the terrible phenomena of disease and death.

---

* Professor Laycock has paved the way to such a course in his Lectures on Medical Psychology in the University of Edinburgh, and his admirable work *Mind and Brain*, although written specifically for the student of medical psychology and the general student of mental science, should be read by every student of medicine whatever, as a masterly exposition of medical philosophy.

